# Erratum

**DOI:** 10.5935/abc.20160075

**Published:** 2016-05

## Issue of March 2016, vol. 106 (3), pp. 168-170

In the editorial "Prophylactic Left Internal Mammary Artery Graft In Mildly-Stenosed Coronary Lesions. Still An Open Discussion", adjust the name of author André Schmidit to André Schmidt.

## Issue of August 2015, vol. 105 (2), pp. 123-129

In the original article "New Exercise-Dipyridamole Combined Test for Nuclear Cardiology in Insufficient Effort: Appropriate Diagnostic Sensitivity Keeping Exercise Prognosis", consider Vidal I as the correct form for the name of the author Inés Vidal Cortinas.

## Issue of May 2016, vol. 106 (5), pp. 373-381

Consider correct and use to citation the author's name Wojciech Wanha to the article "First- Versus Second-Generation Drug-Eluting Stents in Acute Coronary Syndromes (Katowice-Zabrze Registry)".

